# Seed Biochemical Composition and Yield of Four Fenugreek Genotypes

**DOI:** 10.3390/biology15141196

**Published:** 2026-07-20

**Authors:** Zoheir Benzekri, Ahmed Adda, Kamel Zemour, Zahreddine Djazouli, Romain Valentin, Eric Lacroux, Othmane Merah

**Affiliations:** 1 Ecole Supérieure des Sciences de l’Aliment et des Industries Agroalimentaires, Oued Smar 16059, Algeria; zoheir.benzekri@gmail.com (Z.B.); zahro2002@gmail.com (Z.D.); 2 Laboratory of Agro-Biotechnology and Nutrition in Semi-Arid Zones, Faculty of Nature and Life Sciences, Ibn-Khaldoun University of Tiaret, Tiaret 14000, Algeria; adda2ahmed@yahoo.fr; 3 Laboratory of Agronomy Environment Research, University of Tissemsilt, Tissemsilt 38000, Algeria; kamel.zemour@univ-tissemsilt.dz; 4 Laboratoire de Chimie Agro-Industrielle (LCA), Université de Toulouse, INRA, INPT, 31030 Toulouse, France; romain.valentin@inp-toulouse.fr (R.V.); eric.lacroux@inp-toulouse.fr (E.L.); 5 Département Génie Biologique, Institut Universitaire de Technologie, Université de Toulouse, 32000 Auch, France

**Keywords:** fatty acid profile, oil content, antioxidant activity, *Trigonella foenum-graecum*, seed, oilcake, genotypic variability

## Abstract

Fenugreek is a legume that has been used since ancient times to treat certain conditions. Numerous studies have examined the seed’s composition in terms of trigonelline, diosgenin and galactomannans, and their implications for nutrition and medicine. The oil content, fatty acid profile and antioxidant activity of fenugreek seeds have been less extensively studied, particularly in certain areas of the Mediterranean basin. The aim of this study was to examine the oil content and composition, as well as the antioxidant activity of the seeds, oil and meal from four genotypes originating from Algeria, France, Lebanon and Syria. The trials were conducted over two consecutive years with contrasting climatic conditions, in the open field, at the University of Tiaret (western Algeria). Climatic conditions significantly affected all studied traits. In 2023, a dry and hot year, the plants produced low grain yield and low oil content compared to 2024, a temperate year, regardless of the genotype. The fatty acid composition was dominated by polyunsaturated fatty acids (PUFAs, between 65.5 and 68.8%). The phenolic and flavonoid contents were higher in 2023 than in 2024, resulting in greater antioxidant activity under hot and dry conditions of 2023. Genotypic differences were also statistically significant. A more varied responses in grain yield were observed for the French genotype. The highest oil content was recorded for the Algerian and Syrian genotypes. The antioxidant activities of the seed and the oil were similar and significantly higher than that of the oilcake. This activity correlated with the levels of phenols and flavonoids. The results observed in this study provide a better understanding of genotypic diversity and the impact of climatic conditions on yield and seeds quality. They also pave the way for the use of these genotypes in larger-scale breeding programs to improve specific traits.

## Simple Summary

Fenugreek is one of oldest and most important Fabaceae for its uses in traditional medicine and pharmaceutics. This species is an important source of galactomannan, saponins and alkaloids. This species has also been studied for its content in lipids. Several studies have reported broad genotypic and environment variability on lipid content. However, there are no reports on lipid content in Lebanon, Algerian, Syrian and French genotypes. This study aims to examine the differences between these genotypes cultivated during two cropping seasons in Algeria. Significative impact of both climatic conditions and genotype has been observed. Seeds’ yield and oil content were higher in early genotypes. Water stress induced a decrease in both agronomic and biochemical traits regardless of the genotype. Higher content in phenol and flavonoid contents was observed in seeds and oil and associated with high antioxidant activities. These results highlighted the interest in oil and its biological activities as potential oil for pharmaceutics uses.

## 1. Introduction

Fenugreek (*Trigonella foenum-graecum* L.), an annual legume, is used extensively in human nutrition. It is among the oldest medicinal plants cultivated worldwide [1,2,3]. Various parts of the plant are used for medicinal and culinary purposes. In ancient times, this species was used as a fodder, pharmaceutical and vegetable plant. The domestication of fenugreek dates back to the Neolithic period in Mesopotamia [4]. It was used to treat gynecological and skin disorders in ancient Egypt in accordance with the prescriptions of the Ebers Papyrus [4]. Early empirical uses are likely due to the seed’s high content of mucilage, diosgenin and trigonelline. The seeds of fenugreek are well-known for their volatile organic compound content. They are known to have cholesterol-lowering and anti-diabetic properties, as well as galactogenic and lactation-promoting effects, biocidal activity, gastric stimulation and hepatoprotective effects, and anti-cancer properties. According to Wani et al. [5], the antidiabetic and cholesterol-lowering effects of fenugreek are primarily attributable to its high dietary fiber content. These seeds are also known for their high protein and gum content [6,7,8,9].

Cutting-edge research has been focusing, increasingly, on trigonelline for its antidiabetic effects, diosgenin for its hormonal applications, and gum as a functional ingredient and its potential applications in the food and pharmaceutical sectors [8,10,11,12,13,14,15,16,17,18,19,20,21]. Lipid composition remains largely underexplored.

Akbari et al. [22] investigated the composition of oil of Malaysian seed. Similar studies have been conducted in Nigeria [23], Bangladesh [24] and Pakistan [25,26]. These studies have used market seeds to determine the oil and fatty acid composition. Saxena et al. [27] have investigated effect of moisture stress on yield and oil composition on thirteen Indian genotypes. They observed that water stress after flowering had a negative impact on grain yield but a positive effect on oil content. Camlica and Yalzik [12] have studied twenty cultivars from different regions under irrigated and stressed field conditions. They succeeded in identifying genotypes rich in alkaloids or saponins. Other genotypes were found to have high oil content. They also highlighted the high antioxidant activity in certain Turkish genotypes and varieties. Ciftci et al. [28] have used nine genotypes to examine oil and fatty acid composition of fenugreek cultivated in Canada. A similar fatty acid profile was observed across the nine genotypes studied, with some variations in the predominant fatty acids. The main lipid antioxidant component in fenugreek was α-tocopherol. More recently, Coban et al. [29] have studied thirty-one genotypes from different geographical origins cultivated during two years in Turkey. They noted that there were significant variations in lipid composition linked to environmental factors (water availability) and genetics. Certain genotypes exhibited profiles of interest in terms of their linolenic or linoleic acid content. In these studies, no genotypes from Algeria, Tunisia and Lebanon have been examined.

Antioxidants are important for the physiological functioning of plants. They have been measured in extracts from seeds genotypes from various geographical origins [12,30]. Wide variability was observed in the content of phenols, flavonoids and antioxidant activities in an acellular system. Khlifi et al. [31] have studied extracts of Tunisian fenugreek seeds for their phenolic and flavonoid content and their biological activity (including antioxidant and antidiabetic activity). Another study showed that the application of nitric oxide or hydrogen sulphide increases the content of secondary metabolites (including phenols and flavonoids) in fenugreek seeds [32]. These studies were carried out on seed extracts using various solvents. The antioxidant activity of seed oil of fenugreek has been studied in one report [22]. It has been proposed to use this oil for pharmaceutical purposes. No study has reported antioxidant measurements for the oil, cake and whole seed concurrently. Furthermore, no systematic study on the oil content or fatty acid composition of fenugreek has been published to date for fenugreek from Algeria, Syria or Lebanon.

The aim of this study is to characterize the agronomic yield and biochemical composition of four genotypes of fenugreek from different geographical origins, grown in field conditions over a two-year period.

## 2. Materials and Methods

### 2.1. Seeds and Experimental Conditions

The seeds were purchased at local markets (Beirut, Lebanon, and Aleppo, Syria) as seeds for cultivation. Certified seeds were bought from seed production companies in Algeria (Prime Agro Irrigation, Boufarik) and France (GSN seeds, Riscle). Sowing was carried out in open fields at the University of Tiaret (Algeria). The site is located east of the town of Tiaret, at an altitude of 925 m, with a latitude of 35°26′37 N and a longitude of 1°38′44 E. The soil is sandy loam, with 2.75% organic matter and a pH of 7.9. The available nitrogen and phosphorus contents are 1.2% and 0.01%, respectively. Applications, at the rate by hectare, of 60 kg of NH_4_NO_3_, 137 of P_2_O_5_ and 75 K_2_O were supplied at sowing. The previous crop on the trial plot was durum wheat. The soil was plowed to a depth of 30 cm and loosened using a disk roller prior to sowing. The sowing was carried out using a cereal seed drill at a depth of 3 cm at the rate of 150 seeds.m^−2^. The soil was firming down after sowing to ensure good contact between the seeds and the soil. There was no significant presence of fungal diseases. Weeds were managed manually each week. The plots for each genotype were harvested manually and separately.

Sowing was carried out on 18 and 16 March in 2023 and 2024, respectively. Flowering took place on 1 and 11 July in 2023 and 2024, respectively. Maturation occurred on 2 and 15 September in 2023 and 2024, respectively. The experimental design used was a completely randomized block design with genotype as the factor, and each genotype was sown with five replicates. Each replicate plot was 12 m^2^. For the biochemical analyses, for each biological replication, the extractions and measurements were performed in triplicate.

### 2.2. Climatic Conditions

The two years differed in terms of climatic conditions (Table 1). The first year, 2023, can be considered a hot, dry year, whereas 2024 was a temperate year. Indeed, in 2024, rainfall was 170 mm higher than in 2023. In 2024, the plants received over 190 mm more water than in 2023 (Table 1). During the grain formation and filling period, the plants received seven times less rainfall in 2023 than in 2024. Average temperatures were 2.3 °C higher in 2023 than in 2024. This difference is more pronounced during the seed formation and filling cycle and exceeded 4 °C (Table 1).

### 2.3. Measurements

#### 2.3.1. Agronomic Traits

The stages of emergence, flowering and maturity were observed. Grain yield (g per plant) was measured at seed maturity on five plants per replicate. Each plant was threshed separately and the seeds were weighed after 48 h of drying at 60 °C to ensure equivalent moisture content at 15%. The average of the five plants for each plot in each block is presented.

#### 2.3.2. Biochemical Analyses

For the biochemical analyses, for each biological replication, the extractions and measurements were performed in triplicate.

Before extraction, the seeds from all samples were dried in a ventilated oven at 100 °C for 24 h until moisture content of 10% was achieved, in accordance with the method described by Uitterhaegen [33].

All reagents were purchased Sigma Aldrich (St Quentin Fallavier, France). Samples of each plot for each genotype were used for extractions with three technical replications.

The oil was extracted using the Soxhlet method with cyclohexane to ensure the recovery of both polar and neutral lipids. The extracted oil was used for the analysis of fatty acid composition. After methylation and conversion to fatty acid methyl esters (FAMEs), methyl tert-butyl ether was added to the oil (1 mL added to 20 mg). One hundred microliters of this mixture were placed on an insert, and 50 µL of trimethylsulfonium hydroxide were then added. The mixture was gently stirred. GC/FID (Perkin Elmer, Norwalk, CT, USA) was used to analyze the FAME, using a 50 m long CP-Select CB capillary column for FAME, made of WCOT-fused silica, with an internal diameter of 0.25 mm and a film thickness of 0.25 µm. The temperature program selected for the analysis was as follows: the oven was held at an initial temperature of 185 °C for 40 min. The temperature then increased to 250 °C at a rate of 15 °C/min and held at 250 °C for 10.7 min. The injector and detector temperatures were the same at 250 °C. Helium was used as the carrier gas. The flow rate and split ratio were 1.2 mL/min and 1:100, respectively.

The oilcake obtained after defatting was oven-dried (60 °C during 48 h) in accordance with ISO 665:2000 [34]. The protein content was determined in accordance with ISO 5883-1:2005 [35], and the conversion factor used to calculate the protein content was 6.25.

The Folin–Ciocalteu method, as modified by Sayed Ahmed et al. [36], was used to assess total phenolic content (TPC). Absorbance was read at 765 nm. TPC was estimated from a calibration curve based on standard solutions of gallic acid in the range of 50–500 mg L^−1^. The TPC was expressed in milligrams of gallic acid equivalent (GAE) per gram of extract (mg GAE g^−1^ extract).

To determine the total flavonoid content (TFC) in the various samples, the aluminum chloride colorimetric method [36,37] was used. The samples were incubated for 15 min, and the absorbance was measured at 510 nm using a UV-visible spectrophotometer. The blank (control) sample consisted of distilled water. A rutin-based calibration curve was used for quantification. The TFC was expressed in mg of rutin equivalents per gram of extract (mg RuE g^−1^ extract).

The method described by Brand-Williams et al. [38] was used to determine the antioxidant activity against the stable DPPH radical. Measurements were carried out using a UV-visible spectrophotometer to determine the absorbance at 515 nm. The measurements were performed at room temperature. Solutions of Trolox in methanol in a concentration range of 100 to 750 μmol/L were used for the standard curve.

The radical scavenging capacity of fenugreek oil was also assessed using the ABTS assay. This was carried out according to the protocol described by Zielinski et al. [39]. Separate solutions of 7 mM ABTS and 2.45 mM potassium persulphate were prepared. These two stock solutions were used to prepare the test solution by mixing equal volumes of each. The mixture was left to stand for 16 h in the dark at room temperature. Subsequently, 150 mL of fenugreek extract was mixed with 2.85 mL of ABTS solution, and absorbance was measured at 734 nm following incubation for two hours in the dark. Methanol was used as a blank, and the oil’s free radical scavenging activity was calculated using the following equation:

$$ABTS\left(\%\right)=\frac{Control\;Abs-Sample\;Abs}{Control\;Abs}\;*\;100$$

Control Abs and Sample Abs represent the absorbances of ABTS and methanol solution without sample and the mixture of oil and ABTS, respectively.

### 2.4. Statistical Analyses

The data obtained in this study were subjected to a test for homogeneity. It was found that the data were homogeneous and followed a normal distribution.

A two-way analysis of variance was conducted on the four genotypes and the two years simultaneously to determine the individual effects and their interaction on the measured traits. Duncan’s test was used to compare the mean values of the traits and between years. A correlation analysis was also carried out to examine the statistical relationships between the traits. A principal components analysis was done to highlight similarities or differences between the measured traits, to identify the traits most strongly correlated, and to produce a graphical representation of the resulting principal components.

## 3. Results

### 3.1. Agronomic Performance and Climate Impact

The genotype, year and their interaction have a significant impact on fenugreek grain yield. In 2024, significantly higher grain yield was observed than in 2023 (Table 2). In fact, the plants produced 60% more grain yield in 2024 compared to 2023, regardless of genotypes. In the latter year, all genotypes yielded less grains than in 2024 (Table 2). However, genotypic behavior was not the same. The Middle Eastern genotypes yielded 0.5 g less per plant in 2023 than in 2024. This contrasted with the French genotype, which produced half as much in the dry year as in 2024.

The year, genotype and their interaction effects statistically influenced the phenological characteristics (Table 2). The growing cycle duration ranged from 127 to 150 days depending on the genotypes and cultivation years. In 2023, fenugreek flowered and ripened earlier than in 2024, regardless of the genotype (Table 2). The genotypes from the southern Mediterranean showed significant precocity. The Algerian genotype was 5 to 10 days earlier than the French genotype, regardless of the year. This precocity was already evident at flowering and became more pronounced as the fruit ripened (Table 2).

### 3.2. Proximate Composition

The genotype, year and their interaction effects significantly influenced all biochemical traits (Table 3). The year 2023 showed the lowest values for lipids, with the exception of protein content, compared to 2024 (Table 3). The oil content was significantly higher in 2024 compared to 2023. A difference of 1.5 and 1.6% of oil between the extreme genotypes was observed in 2024 and 2023, representing more than a quarter of the oil content. The highest content was recorded for the Syrian and Algerian genotypes, whilst the lowest was observed for the French genotype.

The fatty acid composition of fenugreek was dominated by unsaturated fatty acids (UFAs), which varied from year to year. Average UFAs, regardless of genotypes, accounted for more than 80.4% and 82.8% in 2023 and 2024, respectively. Polyunsaturated fatty acids (PUFAs) accounted for more than a third of total fatty acids (Table 3). Linoleic and linolenic acids alone accounted for 2/5 and 1/4 of the fatty acids in fenugreek, respectively. In 2023, these PUFAs and UFAs were 2.4% lower than their level in 2024. Conversely, saturated fatty acids (SFAs) followed the opposite trend. These SFA values were 2.6% higher in 2023 compared to 2024 (Table 3). This trend is observed across all genotypes, with the impact of annual climatic conditions varying. The smallest differences between years for SFA, PUFA and UFA were observed for the Algerian genotype. The other three origins showed broadly similar changes between 2023 and 2024 (Table 3).

A significant increase in protein content was observed in 2023 compared with 2024. This difference was noted across all genotypes (Table 3).

The year, genotype and their interaction significantly influenced the phenolic and flavonoid contents and the antioxidant activities of the seeds, oil and oilcake (Table 4). The seeds exhibited the highest TPC, TFC, DPPH and ABTS values. The values obtained for the oil, although slightly lower, were quite similar to those of the seed and higher than those of the oilcake (Table 4). The highest antioxidant content and antioxidant activity values (both DPPH and ABTS) were observed in 2023 regardless of genotype and substrate (Table 4). Similarly, the highest TPC and TFC levels and antioxidant activity were observed for southern genotypes regardless of the substrate used for extraction or the year.

**Figure 1:**
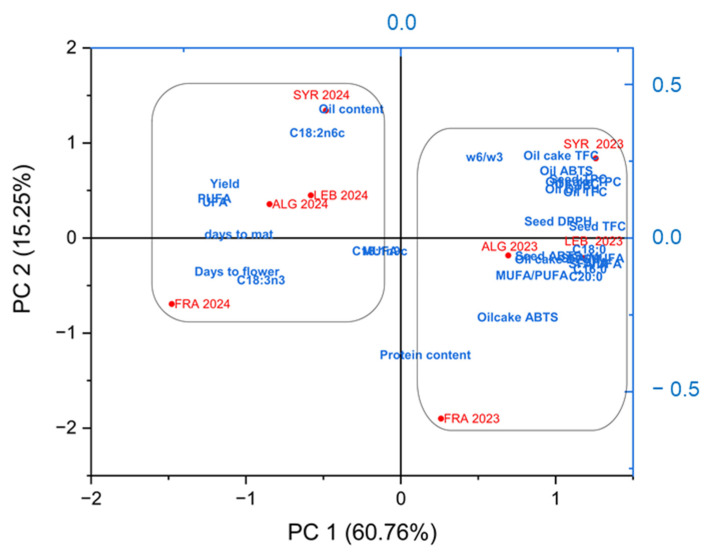
Principal component analysis based on measured traits on four genotypes from different origins cultivated at University of Tiaret (Algeria) during two cropping seasons (2023 and 2024).

Principal component analysis (Figure 1) showed that the first two vectors accounted for more than 76% of the observed variability. The first component was positively correlated with fatty acid composition, phenols, flavonoids and antioxidant activity, and negatively related to grain yield, phenology and unsaturated fatty acid content. The second component was positively correlated with oil content and negatively correlated with protein content. Two homogeneous groups emerged, linked to the two years covered by the study. However, within each group, there was a contrast between the French genotype and the Syrian one, and to a lesser extent with the other two genotypes (Algeria and Lebanon), regardless of the year.

**Figure 2:**
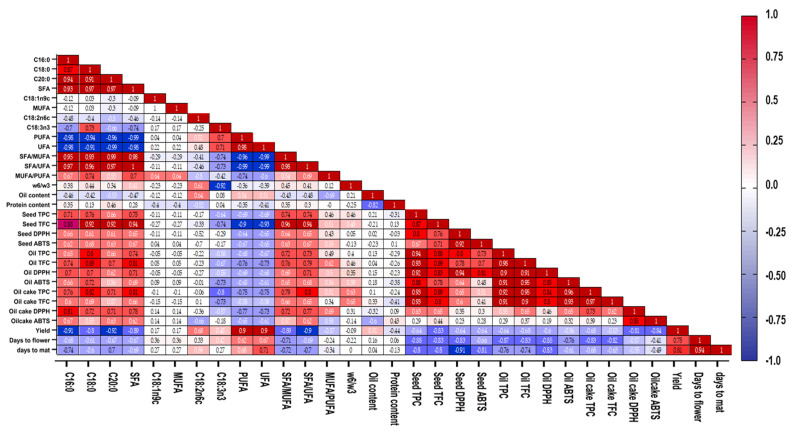
Correlation coefficients between measured traits on four genotypes from different origins cultivated at University of Tiaret (Algeria) during two cropping seasons (2023 and 2024).

Correlation analysis (Figure 2) showed that, in addition to the expected relationships between the various fatty acids, the protein content was negatively correlated with the oil content (Figure 1 and Figure 2). TPC and TFC were positively correlated with antioxidant activity regardless of the substrate (seeds, oil or oilcake). Furthermore, these biochemical traits were negatively correlated with phenology and grain yield, highlighting that the latest-maturing and most productive genotypes were those with the lowest levels of phenols and flavonoids and the lowest antioxidant activity (Figure 2).

Grain yield and phenology were negatively correlated with saturated fatty acid content and positively correlated with polyunsaturated fatty acid content. The latest-maturing genotypes produced the highest grain yields (Figure 2).

## 4. Discussion

### 4.1. Agronomic Performance and Climate Impact

Fenugreek, an ancient and versatile medicinal legume, is used for its medicinal properties across multiple cultures [13,39]. It can be used as a nutraceutical, a functional food, animal feed or even as a cover crop for ecosystem services [11,24,40,41]. The aim of this study was to investigate the agronomic traits and biochemical composition of four fenugreek genotypes from different geographical origins, grown in the open field over two years.

Seed yield varied between the two growing years and among the fenugreek genotypes grown (Table 2). The average yield across all genotypes and both years was 4.14 g per plant. This average value is nearly four times higher than that found by Sana et al. [32] when examining the effect of signaling transmitters in fenugreek in India. Meena et al. [42] studied the diversity of morphological and agronomic traits in 204 fenugreek accessions over three growing seasons in Jodhpur, India. The average seed yield was around 25 g per plant. These results contrast sharply with our findings. These findings can be explained by two hypotheses. The first relates to the differences between the genotypes studied and the climatic conditions prevailing during the growing season. The second hypothesis, which is the most probable, is related to the measurements themselves. It is possible that in this latter study, the yield was measured across plot harvesting, while in our study we have done five randomly selected plants in central rows of 12 m^2^ plot. Our results are consistent with those of Saxena et al. [27], who examined 13 genotypes under water-stress conditions. Other studies have shown variability in fenugreek yield [12,42,43,44,45]. By considering the sowing density (150 seeds per m^2^) and germination rate (80% in our case) we have calculated the yield by square meter. The obtained yield was 381.6 and 607.2 g per square meter for 2023 and 2024, respectively. These results are two times higher than those observed by Meena et al. [42]. Camlica and Yalzid [12] also observed a decrease in yield between irrigated and rainfed trials, which is consistent with our results.

The grain yield in 2023 was nearly 40% lower than in 2024 (Table 4). One of the main reasons for the difference in grain yield between years and genotypes is the variation in rainfall amounts during the growing season and between the vegetative and reproductive-maturation stages [27,42]. In our study, the differences in rainfall and temperature between 2023 and 2024 were very significant throughout the growing season (Table 1). The year 2023 was a dry and hot year, in contrast to 2024, which was a relatively wet and temperate year (Table 1). The fenugreek plants received seven times less water during the flowering-maturation period in 2023 than in 2024. Our results, however, are within the range of data reported by Saxena et al. [26], who examined the effect of water stress at different growth stages on seed yield and oil yield in 13 genotypes grown in India. In the study of Saxena et al. [27], it has been observed that yield was negatively affected by stress occurring at post flowering stage.

Significant differences between genotypes were observed in terms of grain yield (Table 2). The French genotype exhibited the greatest variation in grain yield and showed the most extreme values over the two years. In contrast, the other genotypes showed the smallest range of variation between the two contrasting growing years (Table 2). This difference is mainly due to variations in phenological cycle duration (Table 2). The French genotype is the latest maturing of the four genotypes studied. In contrast, the Algerian genotype is the earliest maturing. In other oil crops, it has been shown that southern genotypes develop a strategy to escape late-cycle thermal and water stress, thereby allowing for better grain filling [46,47].

As for grain yield, genotypes and climatic conditions had a significant impact on oil content (Table 3). The mean oil content values are higher than those reported for fenugreek seeds in Saudi Arabia and Malaysia [22,48], and lower than those observed in Sudan [49]. However, in those studies, the seeds analyzed were purchased from markets. Our results fall within the range (5.8–15.2%) obtained by Ciftci et al. [28] when examining nine genotypes grown under the same agronomic conditions. Similarly, the range (6.58% to 9.29%) obtained by Camlica and Yalzid [12] is consistent with our results. In the latter study, 20 genotypes were examined under two water regimes (dry and irrigated). The observed differences in oil content are likely due to either environmental factors or growing conditions as previously reported in the literature [12,29,46]. Other reports have shown impact of solvent on oil content extraction. Ben Abdennebi et al. [50] have shown that cyclopentyl methyl ether yielded 50% more oil than hexane, thus highlighting the impact of the solvent used on the extraction yield of fenugreek oil. Furthermore, no study has examined the oil content of Algerian, Lebanese or Syrian fenugreek. Furthermore, this content was lower in 2023 compared to 2024 (Table 3). This difference is mainly due to the climatic conditions of the two years in our study (Table 1). The year 2023 was hotter and drier than 2024, a temperate year. During the crucial seed-filling period, when oil is synthesized and stored, fenugreek plants received only 4.4 mm of rainfall in 2023, whereas in 2024, the plants received seven times more water. It has been reported that drought, heat and water shortage have an impact on the oil content of fenugreek seeds [12,29].

Linoleic acid (ω-6) is the main fatty acid present in fenugreek oil and is also a major component of the human diet. As a structural component of cell membranes, this fatty acid influences their function and serves as a substrate for the production of eicosanoids. These play a role in respiratory function, inflammatory responses and modulate renal activity [51,52]. Oils with a linoleic acid content exceeding 22% are considered to be siccative [12,53]. They can be used in various cosmetic industries (varnishes, lacquers), inks and paints. The four genotypes in our study can be regarded as a source of siccative oil given their high linoleic acid content (Table 3). Furthermore, the PUFA content was over 65% (Table 3). It is well-known that PUFAs can help prevent cardiovascular disease and diabetes [51,53]. Thus, all genotypes in our study have high PUFA content and can provide these health benefits.

The protein content varied between 27.8 and 30.1 depending on the genotypes and the years of the study. These values are higher than those reported in Saudi Arabia [48], Ethiopia [24] or India [54]. This variation is mainly due to climatic differences between the two years and between genotypes [46,55,56].

Furthermore, the Algerian, Lebanese and Syrian genotypes were richer in oil than the French one. This can probably be explained by several factors. The first relates to the use of fenugreek seeds in the four countries. Indeed, the three oil-rich genotypes are used in human food and for folk medicine purposes [8,10,50,57]. The French genotype, on the other hand, is used as a cover crop in agriculture. Selection has probably indirectly influenced the composition of the seed. This finding may be supported by the higher protein content in the French genotype, regardless of the year of cultivation, compared to the other genotypes studied. The second possible explanation, and the most likely one, relates to phenology. The French genotype matures later than the other three. As a result, it would have been more severely affected by climatic conditions during the growing season. Indeed, the French genotype had an average delay until maturity of nearly 6 and 8.5 days compared with the other genotypes in 2023 and 2024, respectively (Table 2). Under these conditions, it would have accumulated more protein than oil (Table 3). This hypothesis is supported by the significantly negative correlation observed in our study (Figure 2). This result suggests that the more stressed the plants were, the less oil they accumulated and the more protein they synthesized. This finding has already been reported in other species [46,55,56].

As expected, the fatty acid composition was dominated by a high content of PUFAs (Table 3). It consisted of 17.8% SFAs, 14.3% MUFAs and 67.3% PUFAs (Table 3). Unsaturated fatty acids accounted for over 81.5%. A similar profile has previously been reported for fenugreek grown in various regions of the world [12,29,48,54]. The fatty acid profile for 2023, a hot and dry year, was characterized by an SFA content 2.6% higher than that of 2024. Conversely, PUFAs and MUFAs were 2.3% and 2.4% lower, respectively, in 2023 compared to 2024 (Table 3). Consequently, the SFA/MUFA and MUFA/PUFA ratios decreased in 2024 compared with 2023. The four genotypes studied showed differences in fatty acid composition, regardless of the year (Table 3). These differences are due both to the intrinsic activity of oil biosynthesis and accumulation and to phenology (Table 2). Differences in cycle duration can result in the crop coinciding with adverse weather conditions. The French genotype is late-maturing (by nearly two weeks) compared to the Algerian genotype. This resulted in the grain-filling phase and the synthesis of proteins and lipids coinciding with hot, dry conditions. (Table 1 and Table 2). As previously reported in the literature, temperature and rainfall (as well as irrigation) have a significant impact on the fatty acid composition of fenugreek [12,27,28,29]. In these studies, larger collections were examined under stress and irrigation conditions. Our results are in accordance with these findings. The content of saturated fatty acids increased under stress conditions, whilst that of polyunsaturated fatty acids decreased (Figure 2). Similar trends have been observed in cumin, rapeseed, sunflower, safflower, coriander, Spanish sage and woad [31,47,56,58,59,60,61]. Differences in oil content and fatty acid composition were observed at four study sites for the Atlas pistachio in Algeria [62,63]. The levels of PUFAs and MUFAs increased along a climatic gradient, with the highest levels of unsaturated fatty acids observed under rainy and temperate conditions. The same trend was observed in safflower grown at different sowing dates. Late sowing has caused a decrease in unsaturated fatty acids [58]. In the fatty acid biosynthesis pathway, saturated fatty acids are synthesized first. Desaturation reactions, which involve numerous enzymes, convert stearic acid into oleic acid, then linoleic acid and finally linolenic acid [64,65]. The increase in PUFAs was the result of higher levels of saturated and unsaturated fatty acids (Table 3), reflecting the fact that climatic conditions influenced desaturase activity, leading to a lower PUFA content in 2023 compared to 2024. The lower UFA content was solely due to a lower linolenic acid value, likely due, probably, to reduced activity of the Δ15 desaturase (Table 3). However, in 2023, the reduction in PUFAs was not only linked to the reduced activity of the Δ15 desaturase, but also to that which mediates the conversion of oleic to linoleic acid via the FAD2 desaturase [64,65]. This resulted in an increase in stearic acid and the maintenance of oleic acid levels (Table 3).

In our study, the ratio of ω-6 to ω-3 fatty acids ranged from 1.59 to 1.89, regardless of the year or the genotype. This ratio had not been reported in the literature for fenugreek. These results confirm that fenugreek is a valuable source of balanced fatty acids for diets. However, this ratio was higher than those observed for several legumes [66,67] and other oil species as *Salvia hispanica* [68], coriander [57], safflower [47,60] or cumin [37].

### 4.2. Phenols, Flavonoids and Antioxidant Activity

As with oil composition, genotype, year and their interaction significantly influenced the levels of phenolic and flavonoid content as well as the antioxidant activity of the seeds, oil and oilcake (Table 4). The seeds exhibited the highest values for TPC, TFC and DPPH. The values obtained in our study (Table 4) were similar to those reported by Camlica and Yalzid [12], who studied twenty genotypes grown under rain-fed and irrigated conditions, as well as those of Singh et al. [45] on a collection of fenugreeks of different colors. Sana et al. [32] also obtained comparable values in a study conducted in India. Elsherif et al. [69] have obtained values similar to our data when examining the effect of nanoparticles on early germination and seedling development. In the study of Elsherif et al. [69], the extracts were prepared from seedlings. However, the values obtained from fenugreek seed extracts in our study are significantly higher than those previously observed [29,70,71,72]. In these studies, extracts were prepared from seeds purchased from local markets without traceability information (age, harvest date, origin, and storage conditions). It is well-known that age and storage conditions strongly influence the composition of seeds and their viability [69].

The flavonoid content ranged from 13.9 to 18.1 mg RE g^−1^ extract, regardless of genotype or year (Table 4). These values fall within the range reported by Sana et al. [31] in India. Similarly, to phenols, our results for flavonoid content were significantly higher than those of Khlifi et al. [31] and Kenny et al. [70].

The antioxidant activity measured by free radical scavenging (DPPH) in fenugreek seed extracts was very high (Table 4). Similar activities have also been previously reported [12,29,71,72]. The TPC, TFC DPPH and ABTS values obtained for the oil are slightly lower than those for the whole seeds. Only one study has assessed these properties in fenugreek oil [21]. Similarly, the antioxidant activity measured by the ABTS assay showed the same trends as those measured with DPPH. The higher antioxidant activities (DPPH and ABTS) observed in both oil and seeds compared to oilcake mirrored that the major part of activities is related to oil components. Our results appeared similar to those observed in the study of Akbari et al. [22].

The TPC, TFC and DPPH values for the oilcake were significantly lower than those for whole fenugreek seeds or fenugreek oil (Table 4). This is the first report on composition of oilcake of fenugreek. There are no comparative studies on these measurements. The results obtained for all substrates indicate that the antioxidant activities appear to be primarily linked to the phenolic compounds and flavonoids present in the oil.

Significant differences were observed between the two years and between genotypes for antioxidant activity traits. It is well-established that climatic conditions, genotypes, solvents, sampling date, agricultural practices and the phenological stage at sampling strongly influence the levels of phenolic compounds and their antioxidant activity in fenugreek [12,29]. The year 2023 was hotter and drier than 2024 (Table 1). These conditions likely led to a defensive response in the plants in response to water stress and high temperatures during the seed formation and filling stages (Table 1). The synthesis of phenolic compounds is promoted under these conditions [12,29]. Moreover, the antioxidant activities were related to the content of phenols and flavonoids (Figure 2). This fact was expected and previously reported in the literature [22,30]. The antioxidant activities are related to oil TPC and TPC (Table 4). The results obtained in this study on four genotypes grown over two years under contrasting climatic conditions confirmed the strong antioxidant potential of fenugreek oil and seeds. Plants with high phenolic and flavonoid contents exhibited high antioxidant activity leading to consider it fenugreek source as healthy oil. Furthermore, the oilcake also exhibits antioxidant activity that could be beneficial for animal feed [40].

## 5. Conclusions

The aim of this study was to examine the yield and the biochemical composition of the seed, oil and meal of four genotypes—which had not previously been studied—grown over two years with contrasting climatic conditions. The results demonstrated the significant impact of the year (climatic conditions) and genotype factors on all the traits measured. Oil and protein content, as well as yield, vary considerably depending on the year and genotype. The Algerian genotype showed relative stability in terms of yield, oil and protein contents. This stability is likely due to its early maturity. In contrast, the French genotype is higher in protein and lower in oil. This composition is undoubtedly the result of its late maturity and its sensitivity to dry and hot conditions. The other two genotypes are more similar to the Algerian one. Antioxidant activity follows the same trend as the phenolic and flavonoid content, regardless of the extraction solvent used. The TPC and TFC of the oil were similar to those of the seed and significantly higher than those of the oilcake, highlighting the value of fenugreek oil as a source of polyunsaturated fatty acids and antioxidants. These results provide insights into the composition of fenugreek seeds from sources that have previously been little studied. It would be interesting to extend this to other geographical origins and launch cross-breeding programs to improve the biochemical characteristics of this species—which has many beneficial properties—in line with targeted objectives.

## Figures and Tables

**Table 1:** Monthly rainfall and mean temperature during two years of fenugreek cultivation at Tiaret (west of Algeria).

Month	2023		2024	
	Rainfall (mm)	Temperature (°C)	Rainfall (mm)	Temperature (°C)
January	72.8	5.37	9.2	4.52
February	41.7	8.27	23.2	5.43
March	11.5	9.91	121.4	9.32
April	0	10.4	81.4	13.58
May	22.6	18.02	20.1	14.62
June	35.4	25.45	10.6	22.13
July	0	28.95	10.6	21.9
August	0	28.9	1.9	24.9
September	4.4	24.23	19.7	23.93
October	7.7	18.87	35.2	17.32
November	14.1	12.08	44.5	9.78
December	25.4	10.17	26	5.6
Year	235.60	16.72	403.80	14.42
Cropping cycle	73.90	20.28	265.70	17.74
March–Sept				
Flowering—maturity	4.40	27.36	32.20	23.56
July to Sept				

**Table 2:** Mean values of yield and phenological traits of four genotypes of fenugreek cultivated during two years at the University of Tiaret (Algeria) in 2023 and 2024.

Trait	Year	Algeria	France	Lebanon	Syria	Mean	Effect		
Yield (g per plant)	2023	3.03 ± 0.05 ^b^	2.94 ± 0.03 ^b^	3.33 ± 0.08 ^a^	3.39 ± 0.08 ^a^	3.18 ± 0.17 ^B^	Genotype (G)	Year (Y)	G x Y
	2024	4.89 ± 0.06 ^c^	5.61 ± 0.10 ^a^	4.65 ± 0.10 ^d^	5.07 ± 0.10 ^b^	5.09 ± 0.35 ^A^	***	***	***
Days to flowering (days)	2023	86.0 ± 0.3 ^c^	91.0 ± 0.4 ^a^	89.0 ± 0.4 ^b^	87.0 ± 0.6 ^c^	88.3 ± 2.2 ^B^	***	***	***
	2024	90.0 ± 0.4 ^b^	96.0 ± 0.4 ^a^	92.0 ± 0.2 ^b^	91.0 ± 0.2 ^b^	92.3 ± 2.6 ^A^	***	***	***
Days to maturity (days)	2023	127.0 ± 0.4 ^b^	137.0 ± 0.5 ^a^	132.0 ± 0.6 ^a^	133.0 ± 0.4 ^a^	131.5 ± 3.1 ^B^	***	***	***
	2024	136.0 ± 0.6 ^d^	150.0 ± 0.6 ^a^	146.0 ± 0.5 ^b^	142.0 ± 0.9 ^c^	143.5 ± 3.6 ^A^	***	***	***

Within each line (comparison between geographical origin), means with same letter are statistically not different according to Duncan’s test at 0.05 probability level. ***: significant at 0.001 probability level. For the column Mean (comparison between years) means with same letter are statistically not different according to Duncan’s test at 0.05 probability level.

**Table 3:** Mean values of oil (%) and protein (%) contents, fatty acid (%) composition of seeds of four genotypes of fenugreek cultivated during two years at University of Tiaret (Algeria) in 2023 and 2024.

Trait	Algeria		France		Lebanon		Syria	
	2023	2024	2023	2024	2023	2024	2023	2024
Palmitic acid (C16:0)	11.86 ± 0.07 ^c^	10.92 ± 0.09 ^e^	12.12 ± 0.10 ^b^	10.81 ± 0.06 ^f^	12.24 ± 0.12 ^a^	11.01 ± 0.10 ^e^	12.11 ± 0.09 ^b^	11.41 ± 0.12 ^d^
Stearic acid (C18:0)	4.96 ± 0.02 ^c^	4.12 ± 0.04 ^f^	5.08 ± 0.06 ^b^	4.18 ± 0.03 ^e^	5.78 ± 0.07 ^a^	4.72 ± 0.05 ^d^	5.86 ± 0.10 ^a^	4.27 ± 0.08 ^e^
Arachidic acid (C20:0)	1.52 ± 0.02 ^c^	1.13 ± 0.01 ^e^	1.64 ± 0.01 ^b^	1.12 ± 0.02 ^e^	1.57 ± 0.03 ^c^	1.18 ± 0.01 ^d^	1.76 ± 0.02 ^a^	1.17 ± 0.01 ^d^
SFA	18.34 ± 0.21 ^c^	16.17 ± 0.19 ^e^	18.84 ± 0.12 ^b^	16.11 ± 0.12 ^e^	19.59 ± 0.13 ^a^	16.91 ± 0.14 ^d^	19.73 ± 0.11 ^a^	16.85 ± 0.12 ^d^
Oleic acid (C18:1n9c)	14.17 ± 0.16 ^b^	14.21 ± 0.11 ^b^	14.19 ± 0.17 ^b^	14.37 ± 0.09 ^b^	14.73 ± 0.15 ^a^	14.56 ± 0.14 ^a^	13.96 ± 0.12 ^c^	14.25 ± 0.13 ^b^
MUFA	14.17 ± 0.18 ^b^	14.21 ± 0.13 ^b^	14.19 ± 0.15 ^b^	14.37 ± 0.11 ^b^	14.73 ± 0.17 ^a^	14.56 ± 0.14 ^a^	13.96 ± 0.12 ^c^	14.25 ± 0.12 ^b^
Linoleic acid (C18:2n6c)	41.57 ± 1.02 ^b^	42.28 ± 1.12 ^ab^	41.52 ± 1.24 ^b^	42.81 ± 1.09 ^ab^	41.52 ± 1.33 ^b^	43.18 ± 0.98 ^a^	43.02 ± 0.99 ^a^	44.21 ± 1.01 ^a^
Linolenic acid (C18:3n3)	25.18 ± 0.72 ^b^	26.52 ± 0.89 ^a^	24.67 ± 0.02 ^c^	25.77 ± 1.02 ^b^	24.03 ± 1.32 ^b^	25.06 ± 1.24 ^b^	22.82 ± 1.43 ^e^	23.87 ± 1.17 ^d^
PUFA	66.75 ± 1.15 ^b^	68.80 ± 1.21 ^a^	66.19 ± 1.07 ^b^	68.58 ± 1.03 ^a^	65.55 ± 1.20 ^c^	68.24 ± 1.06 ^a^	65.84 ± 1.23 ^c^	68.08 ± 1.01 ^a^
UFA	80.92 ± 1.27 ^c^	83.01 ± 1.34 ^a^	80.38 ± 1.24 ^c^	82.95 ± 1.27 ^b^	80.28 ± 1.31 ^c^	82.80 ± 1.11 ^b^	79.80 ± 1.09 ^d^	82.33 ± 1.18 ^b^
Total	99.26 ± 1.34	99.18 ± 1.38	99.22 ± 1.37	99.06 ± 1.40	99.87 ± 1.43	99.71 ± 1.51	99.53 ± 1.41	99.18 ± 1.45
SFA/MUFA	1.29 ± 0.02 ^b^	1.14 ± 0.02 ^c^	1.33 ± 0.01 ^b^	1.12 ± 0.02 ^c^	1.33 ± 0.02 ^b^	1.16 ± 0.01 ^c^	1.41 ± 0.03 ^a^	1.18 ± 0.03 ^c^
MUFA/PUFA	0.21 ± 0.01 ^a^	0.21 ± 0.01 ^a^	0.21 ± 0.01 ^a^	0.21 ± 0.01 ^a^	0.22 ± 0.01 ^a^	0.21 ± 0.01 ^a^	0.21 ± 0.01 ^a^	0.21 ± 0.01 ^a^
ω6/ω3	1.65 ± 0.01 ^c^	1.59 ± 0.03 ^d^	1.68 ± 0.04 ^c^	1.66 ± 0.03 ^c^	1.73 ± 0.04 ^b^	1.72 ± 0.04 ^b^	1.89 ± 0.05 ^a^	1.85 ± 0.03 ^a^
Oil content (%)	6.4 ± 0.11 ^c^	7.3 ± 0.12 ^a^	5.1 ± 0.14 ^e^	5.9 ± 0.13 ^d^	6.0 ± 0.10 ^d^	6.7 ± 0.09 ^b^	6.5 ± 0.12 ^bc^	7.4 ± 0.17 ^a^
Protein content (%dry matter)	29.01 ± 0.17 ^c^	28.12 ± 0.20 ^e^	30.07 ± 0.17 ^a^	29.14 ± 0.15 ^b^	28.17 ± 0.20 ^e^	27.78 ± 0.18 ^f^	28.65 ± 0.019 ^d^	27.98 ± 0.22 ^e^

SFA, MUFA, PUFA, UFA represent saturated, monounsaturated, polyunsaturated and unsaturated fatty acids, respectively. Within each line means with same letter are statistically not different according to Duncan’s test at 0.05 probability level.

**Table 4:** Mean values of total phenolic (mg GAE 100 g^−1^ extract) and flavonoid (mg RuE 100 g^−1^ extract) contents, DPPH (%) and ABTS (mg TE g^−1^ extract) antioxidant activities of seeds, oil and cake of four genotypes of fenugreek cultivated during two years at University of Tiaret (Algeria) in 2023 and 2024.

Trait	Origin							
	Algeria		France		Lebanon		Syria	
Year	2023	2024	2023	2024	2023	2024	2023	2024
Seed TPC	40.12 ± 0.53 ^b^	38.95 ± 0.48 ^bc^	37.82 ± 0.67 ^c^	34.67 ± 0.57 ^d^	41.72 ± 0.82 ^a^	38.16 ± 0.46 ^c^	42.21 ± 0.64 ^a^	39.01 ± 0.71 ^b^
Seed TFC	16.67 ± 0.27 ^b^	14.58 ± 0.24 ^d^	15.87 ± 0.32 ^c^	13.91 ± 0.27 ^e^	16.94 ± 0.48 ^b^	14.78 ± 0.28 ^b^	18.12 ± 0.71 ^a^	14.97 ± 0.65 ^d^
Seed DPPH	191.6 ± 2.78 ^b^	178.7 ± 3.87 ^b^	168.95 ± 2.89 ^b^	163.81 ± 2.83 ^b^	192.34 ± 4.11 ^b^	160.27 ± 3.11 ^b^	185.32 ± 2.11 ^b^	170.11 ± 2.12 ^b^
Seed ABTS	154.78 ± 3.87 ^b^	147.38 ± 3.49 ^b^	141.26 ± 2.21 ^b^	139.52 ± 3.17 ^b^	161.14 ± 3.98 ^b^	133.67 ± 3.54 ^b^	152.53 ± 3.51 ^b^	131.68 ± 3.94 ^b^
Oil TPC mg g^−1^ oil	40.56 ± 0.98 ^c^	36.87 ± 0.72 ^e^	35.47 ± 0.96 f	33.71 ± 0.68 ^g^	41.12 ± 0.78 ^b^	37.84 ± 1.02 ^d^	42.07 ± 0.99 ^a^	36.57 ± 0.87 ^e^
Oil TFC mg g^−1^ oil	15.28 ± 0.92 ^a^	13.42 ± 0.77 ^c^	13.51 ± 0.78 ^c^	12.56 ± 0.69 ^d^	15.89 ± 0.82 ^a^	14.25 ± 0.71 ^b^	15.78 ± 0.69 ^a^	13.98 ± 0.89 ^bc^
Oil DPPH	181.4 ± 2.87 ^b^	167.2 ± 3.10 ^c^	159.68 ± 2.64 ^d^	154.47 ± 3.01 ^e^	187.2 ± 2.56 ^a^	158.7 ± 2.89 ^d^	182.6 ± 3.18 ^b^	169.9 ± 2.89 ^c^
Oil ABTS	156.49 ± 1.89 ^c^	135.71 ± 2.01 ^f^	137.96 ± 2.81 ^f^	129.12 ± 2.65 ^g^	161.12 ± 1.87 ^a^	149.83 ± 1.91 ^e^	159.22 ± 2.00 ^b^	151.84 ± 2.23 ^d^
Cake TPC	21.78 ± 0.46 ^c^	19.23 ± 0.51 ^e^	20.11 ± 0.72 ^d^	17.65 ± 0.38 ^f^	22.41 ± 0.31 ^b^	20.78 ± 0.47 ^d^	23.25 ± 0.28 ^a^	21.09 ± 0.36 ^c^
Cake TFC	10.98 ± 0.13 ^c^	9.87 ± 0.09 ^d^	9.56 ± 0.11 ^d^	8.11 ± 0.09 ^e^	11.23 ± 0.07 ^b^	10.68 ± 0.08 ^c^	12.28 ± 0.15 ^a^	10.87 ± 0.12 ^c^
Cake DPPH	20.15 ± 0.07 ^b^	18.98 ± 0.08 ^d^	21.11 ± 0.07 ^a^	17.97 ± 0.10 ^e^	21.01 ± 0.11 ^a^	20.30 ± 0.12 ^b^	20.24 ± 0.10 ^b^	19.85 ± 0.07 ^c^
Cake ABTS	13.98 ± 0.03 ^b^	13.01 ± 0.02 ^d^	14.52 ± 0.03 ^a^	12.68 ± 0.06 ^f^	13.89 ± 0.04 ^b^	13.74 ± 0.02 ^b^	13.27 ± 0.04 ^c^	12.99 ± 0.04 ^e^

Within each line means with same letter are statistically not different according to Duncan’s test at 0.05 probability level. GAE: gallic acid equivalents, RuE: rutin equivalents, TE: trolox equivalents.

## Data Availability

The data presented in this study are available on request from the corresponding author due to another manuscript preparation.
